# HIV Serostatus Disclosure Among Men Who Have Sex with Men in China in the Era of U=U and PrEP

**DOI:** 10.1007/s10461-021-03502-7

**Published:** 2021-10-25

**Authors:** Wangnan Cao, Jinghua Li, Shengzhi Sun, Carla Sturm, Liping Peng, Jing Gu, Chun Hao, Fengsu Hou, Dannuo Wei, Xinyi You, Yang Deng, Anna Mia Ekström

**Affiliations:** 1 Department of Social Medicine and Health Education, School of Public Health, Peking University, Beijing, 100191 China; 2 School of Public Health, Sun Yat-Sen University, North Campus, 74# Zhongshan 2nd Road, Guangzhou, 510000 China; 3 Sun Yat-Sen Global Health Institute, Sun Yat-Sen University, Guangzhou, China; 4 Department of Environmental Health, Boston University School of Public Health, Boston, MA USA; 5 Department of Global Public Health, Karolinska Institutet, Stockholm, Sweden; 6 Department of Public Mental Health, Kangning Hospital, Shenzhen, Guangdong China

**Keywords:** HIV, Men who have sex with men (MSM), Serostatus disclosure, Undetectable = Untransmittable (U=U), Treatment as prevention (TasP), Pre-exposure prophylaxis (PrEP), China, Antiretroviral therapy

## Abstract

Given the recent evidence on “Undetectable = Untransmittable” (U=U) and pre-exposure prophylaxis (PrEP), the present study aimed to investigate HIV disclosure behaviors and their associations with sexual risk behaviors and U=U and PrEP awareness among men who have sex with men (MSM) in China. A cross-sectional survey was conducted among 689 MSM recruited through a gay-friendly non-governmental organization located in Chengdu, China in 2018–2019. Information was collected by a structured self-administrated questionnaire. The enrolled sample included 554 (80.4%) participants who were HIV-negative and 135 (19.6%) participants with an unknown HIV status. In terms of disclosure, 41.4% of participants informed all partners about their HIV status all the time (informing behavior), while 30.4% asked all partners about their HIV status all the time (asking behavior). Only one-fifth knew about U=U, but this was not statistically associated with either informing or asking behavior. Half (50.5%) had heard of PrEP but this was not statistically associated with either informing or asking behavior. Common barriers to informing and asking behaviors were lower risk perception of HIV infection, a history of sexually transmitted infections, engagement in receptive sex, and a history of sex with casual partners. We found that both U=U and PrEP awareness and HIV serostatus disclosure were infrequent and not associated in this study of Chinese MSM. These data indicate huge information gaps among MSM in China.

## Introduction

Disclosure of HIV serostatus may bring both advantages and disadvantages. Potential advantages include improved adherence to antiretroviral therapy (ART), psychological relief, and shared decision-making with partners in terms of sex and condom use. Potential disadvantages include stigma and discrimination, voilence, and ending of relationships. Whether or not to disclose one’s HIV serostatus is a decision to make after balancing these advantages and disadvantages.

Disclosure of HIV serostatus has been an issue particularly for men who have sex with men (MSM) [1–3], who experience a disproportionate burden of HIV infection [4]. The HIV prevalence among MSM ranges from 5 to 32% globally [5, 6], and has increased among Chinese MSM from 1% in 2005, 7% in 2012, and 10% in 2017 [4, 7–9]. Studies have identifed multiple level of factors associated with HIV disclosure among MSM, including individual factors (e.g., age, illness severity, awareness of HIV status, treatment status), interpersonal factors (e.g., type of the partner, intimacy), and social factors (e.g., social support, stigma against people living with HIV as well as homophobia) [10–12]. The prevalence of HIV disclosure varies from 12 to 53% among MSM living with HIV [13].

Awareness of Undetectable = Untransmittable (U=U) might reduce the need and motivation to disclose HIV status to partners because it is now established that there is no risk of HIV transmission when the viral load is undetectable [14–18]. The 2019–2020 Positive Perspectives Study reported a very high overall awareness of 88% for U=U, regardless of sexual orientation, among people living with HIV in 25 countries including China (50 out of the total 2389 participants), where stigma and discrimination related to lesbian, gay, bisexual and transgender (LGBT) and against people living with HIV is common [19, 20]. However, to our knowledge, there are no studies on the awareness of U=U among MSM in China, nor on whether U=U influences HIV disclosure behavior.

Pre-exposure prophylaxis (PrEP) offers an excellent option for HIV prevention among MSM at high risk of HIV infection [21, 22]. China supports PrEP usage among MSM but has not been integrated into a nation-wide program [23]. MSM in China show interest in PrEP but also express concerns of its side effects, associated stigma, and costs. The awareness of and access to PrEP might reduce the need and motivation to discuss HIV status with partners because they may assume it is the partner’s responsibility to use PrEP to protect them from HIV infection. To the best of our knowledge, no previous studies have been conducted among MSM in China to explore if PrEP awareness and/or usage could affect HIV disclosure behavior.

This study aimed to investigate the prevalence and emerging factors associated with HIV serostatus disclosure among MSM living in Chengdu, China in the era of U=U and PrEP. Tangling these associations would increase awareness on highly effective HIV preventive strategies (U=U and PrEP) and inform MSM to make reasonable and beneficial decisions in terms of HIV disclosure. Along with other potential determinants of disclosure (e.g., risk perception, substance use, condom use), we hypothesized that awareness of U=U and PrEP would be associated with HIV serostatus disclosure behaviors among MSM in the Chinese setting where people living with HIV and sexual minorities are both stigmatized.

## Methods

### Study Design and Setting

We conducted a cross-sectional survey between November 2018 and April 2019 among MSM living in Chengdu, China. Chengdu was chosen as the study site because it is a city in western China presenting a higher than average HIV prevalence among MSM for decades [24]. At the time of the study, PrEP was not covered by any health insurance. The market price of PrEP was about US $290 per month, corresponding to 30% of an average monthly salary among residents living in urban Chengdu [25].

### Participants and Recruitment

Participants were recruited among users of a local gay-friendly non-governmental organization (NGO) whose customers had previously agreed to be contacted for a research purpose. The research team contacted all potential participants from the center’s list of customers by phone to screen for eligibility (assigned male sex at birth, aged 18 years or older, and reported having engaged in anal intercourse with at least one man in the previous six months). A total of 890 individuals were found to be eligible and were invited to visit the NGO to complete a digital self-administered anonymous questionnaire, but 170 individuals declined due to a busy schedule, nconvenience of transportation or concerns on privacy. The remaining 720 individuals agreed to come, of which 711 participants completed the survey in person (response rate of 79.9%) during the study period. Two male experienced fieldworkers from the collaborating NGO were hired to facilitate the screening and recruitment process. A trained research assistant (a graduate student major in Public Health) from the research group conducted anonymous data collection and quality control.

Before completing the anonymous self-administered survey, participants were briefed about the study purpose and were asked to provide written informed consent. The questionnaire took an average of 20 minutes to complete with a table computer (an iPad), and completeness and logic errors were automatically checked. Participants were reimbursed US $8 in cash to compensate for their time spent on the study. Ethical approval was obtained from the Ethics Committee of Sun Yat-sen University ([2018] 049).

### Measures

All survey questions used in the survey were pilot-tested on 15 college students, three MSM peer leaders from the center, and 25 eligible MSM volunteers (who were not invited for the subsequent formal survey). Minor revisions were made based on the pilot results and comments from participants.

#### Background

Socio-demographic information collected included age, ethnicity, education, marital status, personal income, employment status, and local residence. Participants were also asked about sexual orientation and the age of first sexual intercourse with another man.

#### HIV Serostatus Disclosure

HIV serostatus disclosure is an interactive process of asking about a partner’s HIV status (asking) and informing the partner about one’s own HIV status (informing). We used two separate items (inform and ask) to assess participants’ disclosure status: “*In the past month, I informed my partners about my own HIV status*” and “*In the past month, I asked my partners about their HIV status*.” Response options included: never, occasionally, half of the time, most of the time, and always. Two separate outcome variables were constructed, including “*Always informing partners my HIV status*” and “Always asking partners about their HIV status.” Participants who responded with “always” were classified as Y = 1, while the rest were classified together as Y = 0. We used these two outcomes as dependent variables and analyzed for independent determinants.

#### Awareness of U=U and PrEP

We asked participants to determine if the statement “*A person with undetectable viral load cannot transmit HIV to others*” was correct. Response options included: correct, wrong, and I don’t know. Those who responded “wrong” or “I don’t know” were combined as having no U=U awareness.

#### PrEP Awareness and Usage

Participants were asked whether they had heard of any types of PrEP (daily oral PrEP, on-demand oral PrEP, and long-acting injectable PrEP) before the survey. Participants who responded affirmatively to any of these three types of PrEP were classified as having PrEP awareness; those who had never heard of any types of PrEP were classified as having no PrEP awareness. For those who had heard of PrEP, we further asked whether they had used (or were using) it; then they were classified as PrEP users or PrEP non-users accordingly.

#### Other Variables

*HIV status and HIV testing.* Participants were asked about their HIV testing history and HIV status (positive, negative, unknown) at the time of the survey. Each participant was offered to take a free HIV test at the center after completing the survey to confirm their HIV status. However, the present analyses used their self-reported data on HIV status because we believe this is more relevant to disclosure. We also asked about their history of sexually transmitted infections (STIs).

*Risk perception of HIV infection.* Participants who were HIV-negative or had unknown serostatus were asked to rate their perceived risk of HIV infection over the next 6 months using a five-point Likert scale from 1 (very high) to 5 (very low). Responses of very high and high were combined into the higher risk perception group, while responses of very low, low, and neutral were combined into the lower risk perception group.

*Positive attitudes towards living with HIV*. We constructed three items to estimate if the participants had positive attitudes towards living with HIV, including “*people living with HIV can be healthy*”, “*immediate treatment is most effective*”, and “*people living with HIV can have a normal life expectancy*.” Responses included agree (score 1) versus disagree (score 0). A summary score was calculated, with a higher score indicating more positive attitudes towards living with HIV.

*Sexual behaviors.* Participants were asked to recall the total number of partners with whom they had had sex in the past month. For each partner, we asked whether the partnership was regular or casual, whether a condom was used (yes, no), whether drugs were used during sex (yes, no), the venue used to find this partner (online, physical setting, both), and the participant’s role in the sexual intercourse with this partner (insertive only, receptive only, both insertive and receptive). Participants who reported having had sex with more than one partners in the past month were classified as having “multiple sexual partnerships.” Participants who did not use condoms with all partners in the past month were classified into “inconsistent condom use”.

### Statistical Analysis

Univariate associations were assessed using binary logistic regression to examine each of the independent variables listed above with the two outcomes of “*Always inform partners my HIV status”* and “*Always asking partners about their HIV status*”. Subsequently, significant variables (p < 0.05) from the univariate analyses and three variables (U=U awareness, PrEP awareness, and self-reported HIV status; regardless of the univariate analyses results) were included in multivariate logistic regression analyses. The measures of association are presented as unadjusted odds ratio (ORu) versus adjusted odds ratio (AOR), with 95% confidence intervals. All statistical analyses were performed using IBM SPSS Statistics (version 25), and two-tailed p < 0.05 was considered statistically significant.

## Results

### Descriptive Characteristics

#### Background Characteristics

Of the 711 participants who completed the survey, 554 reported being HIV-negative, 135 reported not knowing their status, and 22 reported being HIV-positive. Due to the small sample size of MSM known to be living with HIV in the present study, we are unable to perform a subgroup analysis by HIV status in terms of predictors of disclosure. Those 22 HIV-positive participants were excluded from the final analyses because people with different HIV status would have different motivations, repercussions, and communication challenges in terms of HIV disclosure.

The characteristics of the 689 participants who were HIV-negative or unknown are presented in Table 1 (Table 1). The mean age of the participants was 30 years (SD = 11), and 57.3% had attended college or above. Half (54.0%) of the participants were single, 13.4% were married to woman, and 26.0% were in a stable relationship with a boyfriend. The majority of the participants self-reported their sexual orientation as homosexual (75.9%), and 19.0% self-reported as bisexual, which was more prevalent among married participants. More than half of the participants (54.9%) reported having had their first homosexual intercourse before age 21 years (Table 1).

**Table 1:** Background characteristics and HIV serostatus disclosure status of the participants (N = 689)

Items	N	%
*Background characteristics*		
*Age* (years)		
≤ 25	311	45.1
> 25	378	54.9
*Ethnicity*		
Han	668	97.0
Others	21	3.0
*Local residence*		
No	43	6.2
Yes	646	93.8
*Highest education obtained*		
Below than university	294	42.7
University or above	395	57.3
*Relationship status*		
Single	3872	54.0
Married to a woman	92	13.4
Having boyfriends	179	26.0
Divorced/widow/others	46	6.7
*Employment status*		
Full time	438	63.6
Part time	30	4.4
Unemployed	221	32.1
*Personal monthly income* (USD)		
< 423	221	32.1
423–845	254	36.9
> 845	214	31.1
*Self-identified sexual orientation*		
Homosexual	523	75.9
Heterosexual	3	0.4
Bisexual	131	19.0
Other	32	4.6
*Age of first homosexual intercourse* (years)		
< 21	378	54.9
≥ 21	311	45.1
*HIV serostatus disclosure status*		
*I asked all partners in the past month about their HIV status*		
Never	94	13.6
Occasionally	150	21.8
Half of the time	66	9.6
Most of the time	167	24.2
Always	212	30.8
*I informed all partners in the past month of my HIV status*		
Never	100	14.5
Occasionally	115	16.7
Half of the time	43	6.2
Most of the time	143	20.8
Always	288	41.8

#### HIV Serostatus Disclosure Status

Just over forty percent (41.8%) of participants always informed their partners about their HIV status, while 14.5% never did. Almost one-third (30.8%) always asked all partners about their HIV status, while 13.6% never asked (Table 1). Combining the two outcomes, 179 participants (25.4%) always asked and informed about HIV status, 57 (8.3%) never informed nor asked about HIV status, and 453 (65.7%) participants were in between. These two disclosure behaviors were correlated with each other (Pearson correlation coefficient: 0.786, p < 0.01).

#### U=U and PrEP Awareness/Usage

Only 20% of participants knew about U=U. Approximately half of the participants (50.8%) had heard about PrEP before the study, and the awareness was 32.7%, 29.8%, and 6.2% for daily oral PrEP, on-demand PrEP, and long term injecting PrEP, respectively. None of the participants had used (or were using) any types of PrEP.

#### Other Disclosure-Related Variables

Around one-fifth (18.3%) of the participants had never tested for HIV, while the rest (81.7%) had tested for HIV at least once. Half of the participants (50.6%) had tested in the past six months (recent HIV testing). The study found that 15.1% of participants perceived a high or very high risk of HIV infection, while 58.5% perceived a low or very low risk. Surprisingly few participants (7.8%) reported any history of STI. The mean score of positive attitudes towards living with HIV was 2.45 (SD = 0.70) out of maximum 3.

In the past month, 21.0% of the participants reported having had no partner, 48.2% reported having sex with one person, and 30.7% reported having sex with more than one partner (defined as being in multiple sexual partnerships). More than half of the participants (73.7%) primarily searched for partners through mobile apps (e.g., blued, jack’d), while 20.2% used physical venues (e.g. bars, baths, parks, parties) only, and 6.1% used both ways. One-third of the participants (36.6%) reported inconsistent condom use with all partners in the past month. Around one-quarter of the participants (22.8%) reported drug use during sex. About half (48.9%) reported insertive sex only, 34.6% reported receptive sex only, and 16.5% reported both. Regarding type of partner, 68.8% reported sex with regular partners in the past month, and 47.8% reported sex with casual partners; thus 25.1% (94/374) of those with regular partners also reported sex with causal ones (Table 2).

**Table 2:** HIV and sexual behavioural characteristics of the participants (N = 689)

Items	N	%
*U=U awareness*		
Yes	138	20.0
No	551	80.0
*PrEP awareness*		
Yes	350	50.8
No	339	49.2
*Self-reported HIV status*		
Negative	554	80.4
Unknown	135	19.6
Lifetime HIV testing	563	81.7
Recent HIV testing (< six months)	360	50.6
A history of STIs (Yes)	54	7.8
*Risk perception of HIV infection*		
Very low	170	24.7
Low	233	33.8
Neutral	182	26.4
High	77	11.2
Very high	27	3.9
*Number of sexual partners*		
0	145	21.0
1	332	48.2
2–3	178	25.8
4–5	34	4.9
Inconsistent condom use (Yes)	199	36.6
Drug use during sex (Yes)	124	22.8
*Sexual roles during anal intercourse*		
Insertive sex only	266	48.9
Receptive sex only	188	34.6
Both	90	16.5
Had sex with regular partners (Yes)	374	68.8
Had sex with casual partners (Yes)	260	47.8
*Venue for finding new partners*		
Apps (e.g., blued, jack’d)	401	73.7
Physical setting (e.g., bar, bath, park, party)	110	20.2
Both	33	6.1

Sexual behavior variables were limited to participants who had at least one partner in the past month (n = 544)

*STI: * Sexual Transmitted Infection, *U=U:* Undetectable = Untransmittable, *PrEP:* Pre-exposure Prophylaxis

### Univariate Logistic Regression Analyses Predicting HIV Serostatus Disclosure

#### Sociodemographic and HIV Serostatus Disclosure

Participants were more likely to always inform all partners about their HIV status if they had completed a higher level of education (ORu = 1.72; 95% CI 1.26–2.35) or were in a relationship with a boyfriend (ORu = 2.10; 95% CI 1.46–3.01, compared to single men). Age, as a continuous variable, was negatively associated with always informing all partners about their HIV status (p < 0.001). Older participants (> 25 years old) were less likely to always inform all their partners about their HIV status than younger participants (ORu = 0.45; 95% CI 0.33–0.61). Similarly, age of first male-male intercourse (as a continuous variable) was negatively associated with always informing all partners about their HIV status (p = 0.031). Participants who reported having their first male intercourse at an older age (≥ 21 years old) were less likely to always inform all partners about their HIV status than those who had their first intercourse with another man at a younger age (ORu = 0.66; 95% CI 0.49–0.90). Participants monthly income was marginally associated with their informing behavior, while sexual orientation (homosexual vs. bisexual) was not.

Compared with participants who were single, participants who were in a relationship with a boyfriend were more likely to always ask all of their partners about their HIV status (ORu = 1.73; 95% CI 1.19–2.52). Age, as a continuous variable, was not significantly associated with always asking all partners about their HIV status. However, older participants (> 25 years old) were less likely than their younger counterparts to always ask all partners about their HIV status (ORu = 0.69; 95% CI 0.50–0.96). Compared with participants who earned < US $423 (equals to CNY3000) per month, those who earned US $423–845 (equals to CNY3000-6000) per month had no statistical difference in always asking all partners about their HIV status, but participants who earned > USD845 (equals to CNY6000) were less likely to always ask all partners about their HIV status (ORu = 0.66; 95% CI 0.44–0.98). Participants’ level of education was marginally associated with their asking behavior while employment status, sexual orientation, and age at first intercourse with another man were not significantly associated with asking behavior (Table 3).

**Table 3:** Univariate and multivariate regression analyses of the components associated with HIV serostatus disclosure

Items	Inform partners about one’s own HIV status		Ask partners about their HIV status	
	ORu (95% CI)	AOR (95% CI)	ORu (95% CI)	AOR (95% CI)
*Sociodemographic*				
*Age* (years)				
≤ 25	1.00	1.00	1.00	1.00
> 25	0.45 (0.33, 0.61)***	0.48 (0.30, 0.78)**	0.69 (0.50, 0.96)*	0.84 (0.52, 1.36)
*Highest education obtained*				
Below than university	1.00	1.00	1.00	1.00
University or above	1.72 (1.26, 2.35)***	1.68 (1.11, 2.53)*	**1.34 (0.60, 1.87)†**	1.27 (0.83, 1.94)
*Relationship status*				
Single	1.00	1.00	1.00	1.00
Married to a woman	0.75 (0.46, 1.21)	0.89 (0.61, 2.35)	0.91 (0.54, 1.53)	0.99 (0.50, 1.98)
Having boyfriend	2.10 (1.46, 3.01)***	1.37 (0.86, 2.19)	1.73 (1.19, 2.52)**	1.23 (0.77, 1.97)
*Employment status*				
Full time	1.00	1.00	1.00	1.00
Part time	0.80 (0.36, 1.74)	0.80 (0.30, 2.15)	1.52 (0.70, 3.28)	1.75 (0.69, 4.46)
Unemployed	1.55 (1.12, 2.15)**	1.19 (0.68, 2.08)	1.49 (1.05, 2.10)*	1.07 (0.61, 1.85)
*Personal monthly income* (USD)				
< 423	1.00	1.00	1.00	1.00
423–845	**0.71 (0.49, 1.02)†**	1.10 (0.62, 1.95)	0.81 (0.56, 1.19)	0.95 (0.53, 1.69)
> 845	**0.71 (0.49, 1.05)†**	0.94 (0.50, 1.77)	0.66 (0.44, 0.98)*	0.71 (0.37, 1.35)
*Self-identified sexual orientation*				
Homosexual	1.00	–	1.00	–
Bisexual	0.94 (0.64, 1.39)		1.27 (0.85, 1.91)	
*Age of first homosexual intercourse* (years)				
< 21	1.00	1.00	1.00	–
≥ 21	0.66 (0.49, 0.90)**	0.68 (0.45, 1.04)	0.90 (0.65, 1.25)	
*U=U and PrEP awareness*				
*U=U awareness*				
No	1.00	1.00	1.00	1.00
Yes	0.90 (0.62, 1.32)	1.03 (0.64, 1.65)	0.86 (0.57, 1.30)	1.05 (0.65, 1.70)
*PrEP awareness*				
No	1.00	1.00	1.00	1.00
Yes	1.39 (1.02, 1.88)*	1.34 (0.91, 1.98)	1.09 (0.79, 1.51)	0.96 (0.64, 1.42)
*HIV-related variables*				
*Self-reported HIV status*				
Unknown	1.00	1.00	1.00	1.00
Negative	0.91 (0.62, 1.33)	0.76 (0.45, 1.29)	1.34 (0.88, 2.05)	1.55 (0.90, 2.67)
*Recent HIV testing* (< 6 months)				
No	1.00	–	1.00	1.00
Yes	1.08 (0.80, 1.45)		1.56 (1.13, 2.16)**	1.46 (1.01, 2.14)*
Positive attitudes towards living with HIV	**1.23 (0.99, 1.54)†**	1.14 (0.86, 1.52)	0.98 (0.78, 1.23)	–
*A history of STI*				
No	1.00	1.00	1.00	1.00
Yes	**0.56 (0.31, 1.03)†**	0.65 (0.31, 1.36)	0.45 (0.22, 0.91)*	0.63 (0.29, 1.39)
*Risk perception of HIV infection*				
High/very high	1.00	1.00	1.00	1.00
Very low/low/neutral	0.65 (0.47, 0.88)**	0.60 (0.40, 0.89)*	0.61 (0.49, 0.86)**	0.61 (0.41, 0.91)*
*Sexual behaviors*				
*Multiple sex partnership*				
No	1.00	–	1.00	–
Yes	0.72 (0.51, 1.02)		0.73 (0.50, 1.07)	
*Consistent condom use*				
No	1.00	–	1.00	–
Yes	0.88 (0.62, 1.24)		1.06 (0.73, 1.54)	
*Drug use during sex*				
No	1.00	–	1.00	–
Yes	0.96 (0.64, 1.45)		1.27 (0.83, 1.94)	
*Sexual role during intercourse*				
Insertive sex only	1.00	1.00	1.00	–
Receptive sex only	0.66 (0.45, 0.96)*	0.48 (0.31, 0.74)***	0.74 (0.49, 1.01)	
Both	0.56 (0.34, 0.92)*	0.49 (0.28, 0.84)**	0.89 (0.52, 1.45)	
*Had sex with regular partners*				
No	1.00	1.00	1.00	1.00
Yes	1.68 (1.15, 2.45)**	1.32 (1.04, 2.11)*	1.92 (1.26, 2.91)***	1.06 (0.57, 1.97)
*Had sex with casual partners*				
No	1.00	1.00	1.00	1.00
Yes	0.50 (0.35, 0.70)***	0.50 (0.29, 0.87)*	0.44 (0.30, 0.63)***	0.55 (0.31, 0.97)**
*Venue to know partners*				
Apps	1.00	–	1.00	1.00
Physical setting	0.99 (0.65, 1.52)		0.92 (0.58, 1.44)	0.86 (0.53, 1.40)
Both	0.87 (0.42, 1.79)		0.36 (0.14, 0.96)*	0.45 (0.16, 1.25)

*STIs:* sexually transmitted infections, *ORu:* univariate odds ratio, *STI:* Sexual Transmitted Infection, *U=U:* Undetectable = Untransmittable, *PrEP:* Pre-exposure Prophylaxis, *AOR:* adjusted odds ratio

^a^Sexual behaviors among participants who had at least one partner in the past month (n = 544)

^†^P < 0.10, *P < 0.05, **P < 0.01, ***P < 0.001

#### Association Between U=U Awareness and HIV Serostatus Disclosure

In univariate analyses, being aware of U=U was not statistically significantly associated with either always informing partners about one’s HIV status (ORu = 0.90; 95% CI 0.62–1.32) or always asking partners about their HIV status (ORu = 0.86; 95% CI 0.57–1.30) (Table 3).

#### Association Between PrEP Awareness and HIV Serostatus Ddisclosure

PrEP awareness was associated with always informing partners about their HIV status in univariate analysis (ORu = 1.39; 95% CI 1.02–1.88). PrEP awareness was not statistically significantly associated with asking partners about their HIV status in univariate analyses (ORu = 1.09; 95% CI 0.79–1.51) (Table 3).

#### Associations Between Other Potential Variables and HIV Serostatus Disclosure

Participants’ self-reported HIV status and recent HIV testing were not significantly associated with always informing partners about their HIV status. Positive attitudes towards living with HIV was marginally associated with always informing partners about one’s HIV status in univariate analysis (ORu = 1.23; 95% CI 0.99–1.54, p < 0.1). A history of STIs was marginally associated with always informing partners about one’s HIV status in univariate analysis (ORu = 0.56; 95% CI 0.31–1.03, p < 0.1) (Table 3).

Participants who had sex with regular partners were more likely to always inform partners about their HIV status (ORu = 1.68; 95% CI 1.15–2.45). Participants were less likely to always inform their partner about their HIV status if they perceived themselves to be at lower risk of HIV infection (ORu = 0.65; 95% CI 0.47–0.88), if they reported receptive sex only (ORu = 0.66; 95% CI 0.45–0.96 vs. those who reported insertive sex only), or reported sex with casual partners in the past 6 months (ORu = 0.50; 95% CI 0.35–0.70). Other variables, such as having multiple sexual partnerships, consistent condom use, drug use during sex, and venue to look for partners were not significantly associated with participants’ informing behavior in univariate analyses (Table 3).

Participants’ self-reported HIV status and positive attitudes towards living with HIV were not significantly associated with always asking partners about their HIV status. Recent HIV testing was significantly associated with always asking partners about their HIV status in univariate analysis (ORu = 1.56; 95% CI 1.13–2.16). A history of STIs was significantly associated with always asking partners about their HIV status in univariate analysis (ORu = 0.45; 95% CI 0.22–0.91) (Table 3).

Participants were more likely to always ask partners about their HIV status if they had sex with regular partners (ORu = 1.92; 95% CI 1.26–2.91). Participants were less likely to always ask about their partners’ HIV status if they perceived themselves to be at lower risk of HIV infection (ORu = 0.61; 95% CI 0.49–0.86) and reported having had sex with casual partners (ORu = 0.44; 95% CI 0.30–0.63). Other variables, such as multiple sexual partnerships, consistent condom use, drug use during sex, sexual role during intercourse, and venue to look for partners were not significantly associated with participants’ asking behavior in univariate analyses (Table 3).

#### Multivariate Logistic Regression Analysis Predicting HIV Serostatus Disclosure

**Fig. 1:**
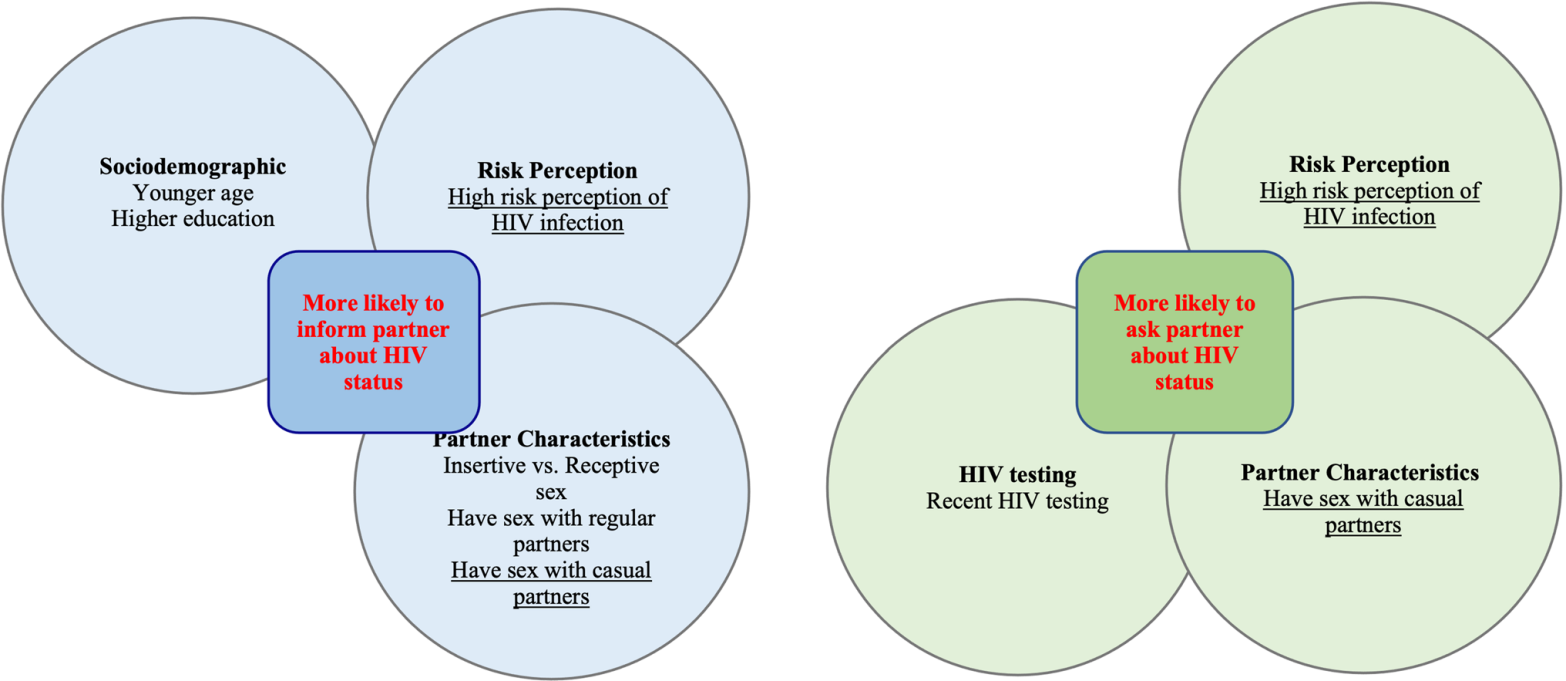
Significant variables for HIV serostatus disclosure among men who have sex with men in Chengdu, China. Variables presented in the figure were statistically significant at the 5% level in multivariate models (Table 3), those underlined variables were significant variables for both informing and asking behaviors.

The variables associated with HIV serostatus disclosure in the multivariate analysis are presented in Table 3 and further illustrated in Fig. 1. Six variables were significantly associated with always informing partners about one’s HIV status, including age (AOR = 0.48; 95% CI 0.30–0.78, > 25 vs. ≤ 25 years old), education (AOR = 1.68; 95% CI 1.11–2.53, university or above vs. below than university), risk perception of HIV infection (AOR = 0.65; 95% CI 0.47–0.88), lower vs. higher risk perception), sexual role during intercourse (AOR = 0.48; 95% CI 0.31–0.74, receptive sex only vs. insertive sex only), having sex with regular partners (AOR = 1.32; 95% CI 1.04–2.11), and having sex with casual partners (AOR = 0.50; 95% CI 0.29–0.87) (Table 3).

Three variables that were significantly associated with asking partners about HIV status all the time: recent HIV testing (AOR = 1.46; 95% CI 1.01–2.14), risk perception of HIV infection (AOR = 0.61; 95% CI 0.41–0.91, lower vs. higher risk perception), and having had sex with casual partners (AOR = 0.55; 95% CI 0.31–0.97) (Table 3).

## Discussion

We found low rates of always asking and informing about HIV status among Chinese MSM. This was not due, however, to high awareness of U=U and PrEP, but rather the opposite because the awareness of treatment as prevention was very low. Half of the participants had tested for HIV in the past 6 months, which was associated with asking more about their partner’s HIV status. Common barriers to HIV disclosure identified in this sample included perceiving a low risk of HIV infection, engaging in receptive sex, and having sex with casual partners. Compared with MSM who did not know their HIV status, HIV-negative MSM reported a similar level of either asking or informing behaviors.

We did not find any association between the U=U awareness and disclosure behavior. The unexpected low level of U=U awareness indicates a huge information gap among Chinese MSM in Chengdu, when compared with MSM in western countries (e.g., Australia, USA) [26, 27], which is in line with Asian men being less aware of U=U compared to Caucasian MSM in a mixed ethnic population in the U.S. [28]. We did not find relevant data for MSM living in other Asian countries for a comparison here. The low awareness might be explained by a lack of advertisements and information about U=U in China, mistrust of the information and its source, and limited availability of viral load testing. In routine HIV care, all patients newly diagnosed with HIV receive ART initiation counseling, in which the counselor mentions U=U as one of the advantages of ART for those who are married or in a stable relationship regardless of sexual orientation. Thus, today, MSM living without HIV would have no or very limited opportunities to get the U=U information from a health professional, i.e., the source of information that most deem as highly credible. Grassroot non-governmental organizations also play a key role in delivering HIV prevention and treatment programs for MSM in China, but they mostly focus on HIV testing and referral for ART. They generally lack skilled professionals, and thereby also the credibility to deliver the U=U information [29]. As a result, awareness of U=U has not spread very widely in the Chinese MSM community. In addition, under the current health and service system in China (Chengdu and elsewhere), viral load testing is provided free of charge only once a year to those on ART for at least 6 months. For most regions, the biggest barrier to viral load testing is the high cost of the assay (US $75 per test) and the lack of sophisticated laboratories and experienced professionals [30].

We did not find any association between PrEP awareness and disclosure behavior. PrEP is offered as an alternative to other HIV preventive strategies and in various forms (daily or on-demand oral PrEP and long-acting injectable PrEP) to meet varying individual needs. Although the hypothetical willingness of PrEP use was high among Chinese MSM if provided for free [23], it is worth noting that PrEP remains inaccessible to most for different reasons, including lack of a supporting national guideline, constrained resources allocated to PrEP, limited trained health professionals in providing PrEP, and high out-of-pocket costs (30% of an average monthly salary) [31, 32]. An increased PrEP awareness may enable MSM to talk more openly about HIV status. However, the association between actual PrEP use and disclosure requires further investigation. We were unable to test this association because none of our participants had any experience of taking PrEP.

A low risk perception of HIV infection was a common barrier to both asking and informing about HIV status, which was consistent with the current disclosure literature among MSM [33, 34]. For example, perceiving a lower risk of HIV infection was associated with less self-disclosure among 1,044 gay and bisexual men in Australia [34]. One approach to deal with inadequate risk perceptions may be to emphasize routine HIV testing [35]. Given that only half of the participants in the current study had tested for HIV in the past 6 months, more efforts are needed to increase access to HIV testing.

MSM engaging in receptive sex were less likely to ask their partners about their HIV status, possibly explained by a lower hierarchical position and less negotiating power in terms of initiating safer sex [36]. However, since receptive MSM have approximately 2 to 6 times higher risk of HIV infection than insertive MSM [37], tailored interventions, such as communication training and empowerment programs, should be provided to all MSM regardless of sexual preference to enhance safer sex behaviors.

The HIV prevalence of 3% in our study was lower than the rates reported among Chengdu MSM in previous studies (e.g., 16% between 2009 and 2014) [38, 39]. The relatively low HIV prevalence might be explained by the recruiting strategy and characteristics of the selected sample. We recruited participants from a local gay-friendly non-governmental organization (Chengdu Tongle Health Counselling Service Center), which mainly provides HIV prevention services and limited HIV treatment services, due to its scope and staff expertise. MSM living with HIV come to the Center to participate in gay community activities and psychological support programs, but most receive their ART at the local hospital and CDC clinic. Thus MSM without known HIV would be more likely to attend the center e.g., for regular HIV testing, making them more likely to be “captured” by our in-person questionnaire. Moreover, the relatively low HIV prevalence could also be explained by the participants being young (half were < 25 years old) and higher educated (56% had a bachelor’s degree) than the average. We found that younger MSM and those with a higher education were more likely than their counterparts to engage in both asking and informing behaviors related to HIV disclosure [40]. Thus, the current disclosure level (30–41%) might be an over-optimistic estimation of the local Chinese MSM population as a whole.

The present study is subject to several limitations. First, participants were recruited through a local NGO, and only MSM who had connections to the local NGO were available for recruitment into this study. Because this NGO has a clientele of MSM that likely are relatively more open about their sexual identity, more educated, have higher incomes, and are younger than average, our findings might not be generalizable to older and less educated MSM or MSM living in more rural areas of China. The present sample also did not include MSM living with HIV. Second, information and recall bias might exist, e.g., with regards to STI history and the age of first homosexual intercourse. STI history was self-reported in the present study, and the prevalence (8.2%) was a bit lower than the data (11.2%) reported at the national level [41], probably due to under reporting or low STI screening in Chengdu. Social desirability bias might also exist regarding questions on sexual behaviors. Third, we constructed our own scale, such as positive attitudes towards living with HIV, which should be validated in future studies. Last, we were unable to recruit MSM who were PrEP users, so the association between PrEP usage and disclosure behaviors could not be determined. Most PrEP users in China have so far received PrEP as part of a research trial, but, to our knowledge, no such trials have been conducted in Chengdu.

Despite these limitations, the current study is the first (to our knowledge) to explore disclosure behaviors in the era of U=U and PrEP in China. The rates of U=U and PrEP awareness were unexpectedly low, 20% for U=U and 51% for PrEP, indicating huge information gaps among MSM in China.

## Conclusion

Overall, HIV serostatus disclosure was low among this young and relatively highly educated sample of Chinese MSM in the era of U=U and PrEP. The awareness of PrEP and U=U was not associated with either informing or asking behaviors. Common barriers to HIV disclosure included perceiving a low risk of HIV infection, engaging in receptive sex, and having sex with casual partners. Having a positive attitude towards living with HIV was associated with always informing partners about one’s HIV status. Those who were older than 25 years old or poorly educated, and those earned less, were less likely to disclose their HIV status and might be in particular need for additional support.

## Data Availability

The data that support the findings of this study are available from the corresponding author upon reasonable request.
